# Immune thrombocytopenia associated with tuberculosis – A case report

**DOI:** 10.1016/j.idcr.2020.e01012

**Published:** 2020-11-19

**Authors:** Adeel Ahmad Khan, Aamir Shahzad, Haleema Javid, Tehreem Fatima, Zohaib Yousaf

**Affiliations:** a Department of Internal Medicine, Hamad Medical Corporation, Doha, Qatar; b Rawalpindi Medical University, Pakistan; c California Institute of Behavioral Neurosciences and Psychology, CA, USA; d Dresden International University, Dresden, Germany

**Keywords:** Immune thrombocytopenia, Tuberculosis

## Abstract

Tuberculosis can involve the hematological system and cause anemia, leucopenia, leukocytosis, thrombocytopenia, and thrombocytosis. Immune thrombocytopenia related to tuberculosis is rare. We present a case of a 54 years old male patient who was evaluated for isolated asymptomatic thrombocytopenia discovered on routine laboratory investigations. Work up was positive for disseminated tuberculosis. The patient responded to a tapering dose of steroids and anti-tuberculous medications with significant and persistent platelet count improvement.

## Introduction

Tuberculosis (TB) is a major communicable. According to the World Health Organization statistics, it is one of the top 10 causes of death worldwide [1]. TB has been associated with a wide range of hematological manifestations, including thrombocytopenia [2]. Immune thrombocytopenia (ITP) as the presenting feature of TB is rare. We present an interesting case of disseminated TB, which presented initially as ITP.

## Case report

A 54-year-old Nepalese gentleman was referred from primary care to evaluate incidental thrombocytopenia (platelet count of 5 × 10^3/uL) found on a routine healthcare evaluation. There was no history of gum bleeding, nosebleed, skin discoloration, easy bruisability, joint pain, blood in stools, blood in urine, fever, weight loss, night sweats, cough, sore throat, nausea, vomiting, or diarrhea. He had ten pack- years of smoking history. He had a history of type 2 diabetes mellitus, hypertension, and ureteric stones. The patient did not have a personal or family history of bleeding disorders, autoimmune diseases, connective tissue diseases, or malignancy. His vital signs were within normal limits. There was no pallor, petechiae, purpura, ecchymoses, skin rashes, or lymphadenopathy on physical examination. Respiratory, cardiac, and abdominal examinations were normal.

**Fig. 1:**
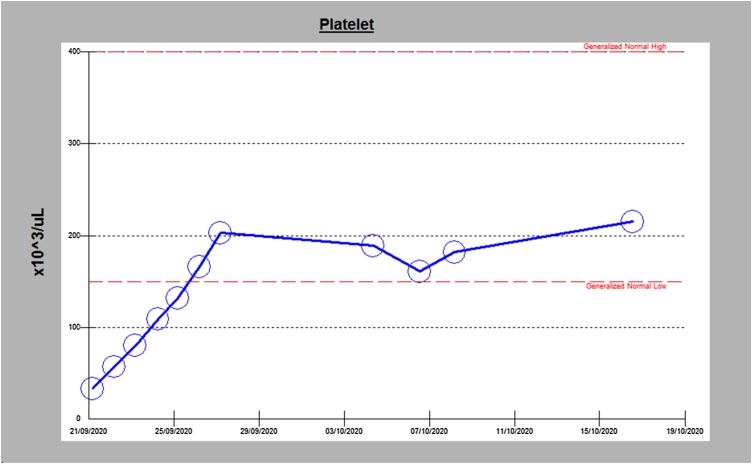
Platelet counts trend.

A complete blood count and serum chemistry, including the anemia profile, was unremarkable except for isolated thrombocytopenia. Peripheral blood smear showed severe thrombocytopenia with normal morphology of blood cells (Table 1). Further workup, including a comprehensive autoimmune screen, viral screening, blood cultures, urine cultures and stool examination for *Helicobacter pylori* antigen, was negative (Table 1). The patient was admitted with a provisional diagnosis of immune thrombocytopenic purpura (ITP) and started treatment with intravenous immunoglobulins for three days and oral prednisolone 10 mg daily. 4 days after starting steroids, the patient's platelet count improved to above 100 × 10^3/uL (Fig. 1).

**Table 1:** General laboratory investigations.

	Lab Values	Normal Range
White blood cells	4.4 × 10^3/uL	4 × 10^3/uL–10 × 10^3/uL
Haemoglobin	12.9 gm/dL	13 gm/dL–17 gm/dL
Platelet count	5 × 10^3/uL	150–400 × 10^3/uL
BUN	4.8 mmol/L	2.8 mmol/L–8.1 mmol/L
Creatinine	72 umol/L	62 umol/L–106 umol/L
ALT	24 U/L	0 U/L–41 U/L
AST	25 U/L	0 U/L–40 U/L
PT	11.5 s	9.4–12.5 s
INR	1.0	1.0
APTT	26.9 s	24.6 s – 31.2 s
LDH	308 U/L	135 U/L–225 U/L
B12	225 pmol/L	145 pmol/L–596 pmol/L

Ultrasound abdomen to rule out splenomegaly showed multiple enlarged lymph nodes with a normal spleen. For further evaluation, a whole-body CT scan was performed. CT thorax showed 3 mm ground glass nodularity in the superior segment of the right lower lung lobe, a fibrotic band in the anterior segment of the left upper lung lobe, and multiple enlarged mediastinal lymph nodes (largest measuring 11 mm). CT scan of the abdomen and pelvis showed a heterogeneous lesion superior to the head of the pancreas. No abdominal lymphadenopathy was noted. MRI abdomen delineate the heterogeneous lesion showed multiple enlarged lymph nodes in the peripancreatic, para-aortic, and aortocaval regions. Based on CT thorax findings, and considering the patient belonged to an endemic area for tuberculosis (TB), a sputum smear for acid-fast bacilli to rule out active pulmonary TB was sent, which was positive. Abdominal lymph node biopsy showed necrotizing granulomatous lesion; no acid-fast bacilli or lymphoma evidence was seen in the biopsy specimen. The patient's ITP was attributed to being secondary to pulmonary TB. Steroids were tapered off, and the patient was started on first-line anti-tuberculosis medications with isoniazid, rifampicin, pyrazinamide, and ethambutol. His platelets continued to improve after stopping the steroids, and he is currently on anti TB medications.

## Discussion

TB can involve the hematological system and can cause anemia, leucopenia, leucocytosis, thrombocytopenia and thrombocytosis. Pulmonary TB is usually associated with thrombocytosis, and disseminated TB is associated with thrombocytopenia [2]. Bone marrow infiltration, hemophagocytic lymphohistiocytosis, thrombotic thrombocytopenic purpura, disseminated intravascular coagulation and immune thrombocytopenia are a few mechanisms attributed to thrombocytopenia development in TB. Moreover, anti TB medications (isoniazid and rifampicin) can also lead to thrombocytopenia [3].

Immune thrombocytopenia is an acquired autoimmune disorder of platelets and is a diagnosis of exclusion. It is characterized by isolated thrombocytopenia with peripheral blood platelet count <100,000/cu.mm, with or without mucocutaneous bleeding [2]. ITP in TB is a rare association. In a study, out of 846 cases of active TB, only nine patients had ITP, out of which, three patients had disseminated TB [4]. Ursavas et al. also described a case of a male patient who presented with typical symptoms of pulmonary tuberculosis and was found to have immune thrombocytopenia [5].

The pathophysiology of TB-related immune thrombocytopenia is unclear. One possible explanation involves the activation of a clone of B lymphocytes by *Mycobacterium tuberculosis* (MTB), leading to antiplatelet antibody production. Another mechanism includes the possibility that MTB and platelets may share antigens with resultant antiplatelet antibody production [2].

Steroid and Immunoglobulins are used to treat adult ITP. Platelet count starts to increase within one week of initiation of treatment. However, in most cases, platelet count decreases again after the withdrawal of steroids, necessitating maintenance steroid therapy. In cases of ITP associated with TB, platelet count does not show response solely to treatment with steroids and Immunoglobulin. However, when treated with a combination of steroids and anti TB medications, platelet count improves. Moreover, in contrast to adult ITP, platelet count does not decrease again with the withdrawal of steroids in patients with TB-related ITP after treatment with a combination of anti TB medications and steroids [6]. Ghobrial et al. described a case of ITP as a presenting manifestation of tuberculosis. Platelet count did not improve solely with IV steroids and IV immunoglobulins. However, after the diagnosis and treatment of TB with anti TB medications, the patient's platelet counts improved, establishing that ITP was secondary to TB [7]. Akyildiz et al. also described a similar case of a child with TB-related ITP [8].

## Conclusion

In patients with immune thrombocytopenia, the possibility of TB-related ITP should be considered, especially in patients belonging to endemic regions. Combination treatment with steroids and anti TB medications results in sustained improvement in thrombocytopenia in such cases.

## Consent

Written informed consent was obtained from patient and is available for provision to the journal on demand.

## Author contribution

AAK wrote the discussion, conducted literature review, and prepared the manuscript. AS wrote the case report section of manuscript and reviewed the manuscript. HJ and TF conducted literature review. ZY critically reviewed the manuscript and contributed to writing discussion.

## Declaration of Competing Interest

The authors report no declarations of interest.
